# Epidemiological studies based on multi-locus sequence typing genotype of methicillin susceptible *Staphylococcus aureus* isolated from camel’s milk

**DOI:** 10.4102/ojvr.v84i1.1425

**Published:** 2017-09-22

**Authors:** Alsagher O. Ali, Hassan Y.A.H. Mahmoud

**Affiliations:** 1 Division of infectious Diseases, Animal Medicine Department, South Valley University, Egypt

## Abstract

One hundred milk samples were collected from camel’s milk for the isolation of *Staphylococcus aureus*. Thirty-one isolates were *S. aureus*, 45 were other forms of staphylococci and 24 represented other bacteria. Five isolates from *S. aureus* were methicillin resistant *S. aureus* (MRSA) and 26 samples were methicillin susceptible *S. aureus* (MSSA). The whole genome sequence of *S. aureus* was annotated and visualised by rapid annotation using subsystem technology (RAST) which is a fully-automated service for annotating complete or nearly complete bacterial genomes. Four isolates from MSSA strains were subjected to multi-locus sequence typing (MLST). Three multi-locus sequences types or sequence types (MLST/ST) were found, namely ST15, ST1153 and ST130. The phylogenetic analysis of the concatenated sequences of the seven genes forming the MLST profile of *S. aureus* classification revealed a high degree of similarity and close relationship between the ST15 and ST1153 while the third ST (ST130) was located in a different cluster.

## Introduction

*Staphylococcus aureus* is a prominent pathogen causing a wide array of diseases in different animal species as well as in human beings. It has been subjected to numerous studies from different countries all over the world, from Egypt (Aly & Abo-Al-Yazeed 2003), Sudan (Shuiep et al. 2009), Saudi Arabia (Zahran & Al-Saleh 1977), Ethiopia (Semereab & Molla 2001), Morocco (Benkerroum et al. 2003; Khedid, Faid & Soulaimani 2003) and the USA (Solyman et al. 2013). Several molecular procedures have been established and used to identify and evaluate *S. aureus* isolates. Coagulase gene (*coa* gene) typing is considered a simple and effective method for typing *S. aureus* isolates from human patients and bovine mastitic milk (Aarestrup, Dangler & Sordillo 1994; Da Silva & Da Silva 2005; Talebi-Satlou, Malahat & Habib 2012; Weese & Van Duijkeren 2010). Epidemiological studies based on analysis of this gene have shown that *S. aureus* isolates could be divided into a number of subtypes (Aslantaş et al. 2007). The multi-locus-sequence typing (MLST) has been used widely to outline the population genetic structure of *S. aureus* as well as other bacterial species. The MLST genotyping could identify different strains, track the spread of methicillin resistance and identify epidemic clones (Holden et al. 2013; Kurt et al. 2013; Nübel et al. 2010). Whole-genome sequencing provides the best discriminatory power between closely related bacterial isolates; hence, because of the rapidly decreasing cost and time required for this technology, it could conceivably become a viable tool in diagnostic laboratories in the near future to reconstruct intercontinental and local transmission of *S. aureus* lineages (Harris, Feil & Holden 2010; Köser, Holden & Ellington 2012).

This present study aimed to investigate the effectiveness of MLST as a method of typing *S. aureus* isolates from camel origin as well as the implementation of multiple sequence alignment and phylogenetic analysis to clarify the molecular characterisation of MLST genes.

## Materials and methods

A total of 100 raw milk samples were collected from dairy she-camels in Red Sea Governorate (Al-shalateen area), Egypt. Animals were examined for body temperature, pulse rate and respiratory rate. Animals were apparently healthy with no local or systemic infection. The milk samples were collected and stored in sterile plastic tubes.

### Isolation and culturing of *Staphylococcus aureus*

*Staphylococcus aureus* was isolated on Baired-Parker media. Three to four typical colonies were harvested and picked up by a sterile metal bacteriological loop then immersed in glycerol and kept at -70 °C – -80 °C for further studies.

### Biochemical tests

The coagulase test was performed by two different methods, namely the slide coagulase test and the tube coagulase test (Field & Smith 1995).

### Detection of *mecA* gene

The *mecA* gene was utilised to affirm the classification of *S. aureus* into methicillin resistant *S. aureus* (MRSA) or methicillin susceptible *S. aureus* (MSSA). The *mecA* gene was analysed by multiplex PCR in addition to the *mecC* gene which was identified recently as a homologue for *mecA*. The *femB* gene was used as a corroborative gene for *S. aureus* (Alsagher 2016; Nakagawa et al. 2005; Paterson et al. 2014).

### Whole genome sequencing and analysis

*Staphylococcus aureus* DNA was used to obtain the genomic sequence of *S. aureus* by shotgun sequencing (Sanger institute, UK).

Artemis was used to manipulate the genome sequence of *S. aureus.* This is a free genome browser and annotation tool that permits visualisation of sequence features, next generation information and the results of investigations inside the context of the sequence and furthermore its six-frame translation (Alsagher 2016; Rutherford et al. 2000). Sequence alignments, translations and comparisons were done utilizing BIOEDIT (Version 7.0.9.0) (Hall 1999). The BLAST algorithm was used to search the NCBI Gen Bank (http://www.ncbi.nlm.hih.gov/) databases for homologous sequences. Neighbour-joining trees (Saitou & Nei 1987) were constructed on the basis of genetic distances and estimated by two-parameter method (Kimura 1980) using MEGA6 (http://www.megasoftware.net) (Tamura et al. 2013). The reliability of the trees was estimated by 500 bootstrap confidence values. To construct the phylogenetic tree, we used 15 STs retrieved from the MLST websites (http://www.mlst.net/) (7, 8, 9, 11, 17, 19,126, 127, 128, 132, 1146, 1147, 1148, 1149 & 1150). In addition, 3 STs (15, 130 & 1153) were extracted from the bacterial chromosome of local isolates. These STs were used to construct the phylogenetic tree.

## Results

Standard culturing methods and the application of coagulase tests for 100 milk samples of she-camels revealed that 31 isolates were *S. aureus*, 45 isolates were other forms of staphylococci, and 24 isolates were other bacteria. According to the presence or absence of the *mecA* gene in *S. aureus* isolates, five isolates were MRSA and 26 were MSSA.

The whole genome sequence of *S. aureus* was annotated by using subsystem technology (RAST) which is a fully-automated service for annotating complete or nearly complete bacterial genomes (http://rast.nmpdr.org/) and Artemis (Rutherford et al. 2000). Four isolates from *S. aureus* were sequenced by the shotgun sequencing technique and the whole genome sequence was obtained. The MLST genes were annotated, extracted and subjected to further analysis to predict the sequence typing by using the MLST websites (http://www.mlst.net/). One local isolated (A11) gave ST 15 (CC 5), local isolates (A12 & A13) gave ST 130 (CC 130) and the local isolate (A15) gave ST1153 (CC 1153) (Table 1).

**TABLE 1:** The sequence similarity and length of multi-locus sequence typing genes of *Staphylococcus aureus* local isolate (A11) and their reference alleles of ST15.

Locus	% identity	HSP length	Allele length	Gaps	Allele
*arcc*	100.00	456	456	0	*arcc-13*
*aroe*	100.00	456	456	0	*aroe-13*
*glpf*	100.00	465	465	0	*glpf-1*
*gmk*	100.00	417	417	0	*gmk_-1*
*pta*	100.00	474	474	0	*pta_-12*
*tpi*	100.00	402	402	0	*tpi_-11*
*yqil*	100.00	516	516	0	*yqil-13*

The seven genes used in *S. aureus* MLST genotyping (yqil, aroe, glpf, gmk, pta, tpi & arcc) were highly polymorphic and they were divided into numerous numbers of different alleles. The allelic composition of each sequence type (ST), similarity and length were shown for ST15, ST130 and ST1153 in (Tables 1, 2 and 3) respectively.

**TABLE 2:** The sequence similarity and length of multi-locus sequence typing genes of *Staphylococcus aureus* local isolate (A12 and A13) and their reference alleles of ST130.

Locus	% identity	HSP length	Allele length	Gaps	Allele
*arcc*	100.00	456	456	0	*arcc-6*
*aroe*	100.00	456	456	0	*aroe-57*
*glpf*	100.00	465	465	0	*glpf-45*
*gmk*	100.00	417	417	0	*gmk_-2*
*pta*	100.00	474	474	0	*pta_-7*
*tpi*	100.00	402	402	0	*tpi_-58*
*yqil*	100.00	516	516	0	*yqil-52*

**TABLE 3:** The sequence similarity and length of multi-locus sequence typing genes of *Staphylococcus aureus* local isolate (A15) and their reference alleles of ST1153.

Locus	% identity	HSP length	Allele length	Gaps	Allele
*arcc*	100.00	456	456	0	*arcc-1*
*aroe*	100.00	456	456	0	*aroe-13*
*glpf*	100.00	465	465	0	*glpf-1*
*gmk*	100.00	417	417	0	*gmk_-1*
*pta*	100.00	474	474	0	*pta_-124*
*tpi*	100.00	402	402	0	*tpi_-5*
*yqil*	100.00	516	516	0	*yqil-3*

The multiple sequence alignment of the translated amino acid sequences of the concatenated MLST genes were shown and aligned with selected other sequence types of *S. aureus* (Figure 2).

**FIGURE 1:**
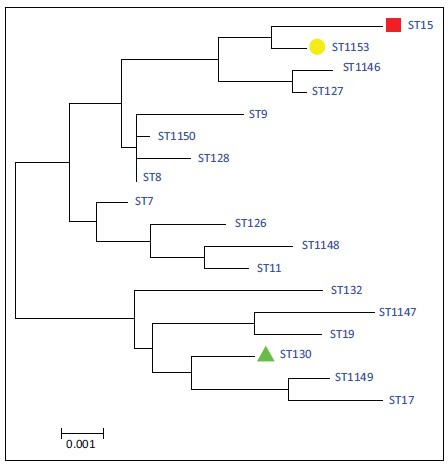
Molecular phylogenetic analysis by maximum likelihood method of multi-locus sequence typing concatenated sequences of *Staphylococcus aureus.*

The evolutionary history was inferred by using the maximum likelihood method based on the Tamura-Neimodel (Tamura & Nei 1993). The analysis involved 18 nucleotide sequences. All positions containing gaps and missing data were eliminated. There were a total of 3186 positions in the final data set. The phylogenetic analysis revealed that ST15, ST130, ST1153 and other sequence types used to construct the phylogenetic tree were divided into two main clusters. The ST15 and ST1153 were closely related to each other and gathered in one cluster away from the third ST130 (Figure 1).

## Discussion

Classification and characterisation of *S. aureus* isolates is an essential and preliminary step to understanding the epidemiological status of this contagious bacterium. Nowadays, MLST genotyping is widely used to investigate the dynamic nature of *S. aureus.* The implementation of the new techniques such as the whole genome sequence can potentially facilitate the mining of bacterial chromosomes for all the *S. aureus* genes. In our study, traditional culturing methods and the coagulase test revealed that 31% of the isolates were *S. aureus*, 45% isolates were other forms of staphylococci and 4% were other pathogens. These results were higher than the 14% reported by Abdel Hameid et al. in 2004. Using the *mecA* gene to classify *S. aureus* isolates into 16.12% MRSA and 83.87% MSSA, our results contradict those obtained by Monecke et al. (2011b) which showed that absence of *mecA* gene and the rarity of antibiotic resistance related genes in *S. aureus* isolates from camel’s milk.

**FIGURE 2:**
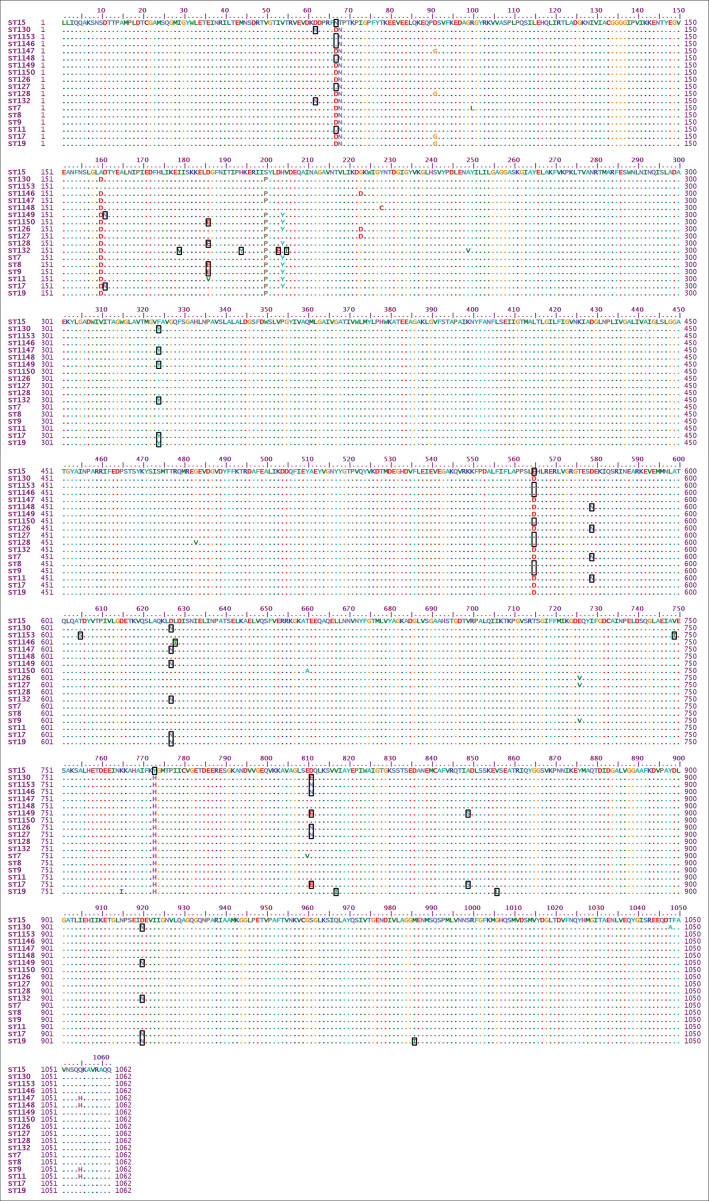
Multiple sequence alignment of amino acids of concatenated sequences of multi-locus sequence typing genes.

The MLST analysis of four isolates of MSSA revealed that three sequence types were recorded (ST15, ST130 and ST1153) in (A11, A12, A13 and A15) local isolates respectively (Table 4, Figures 1 and 2). The presence of ST15 in MSSA isolates was in agreement with another study in livestock and humans (Sanz 2013). ST130 was found in two local isolates (A12 and A13), both of which were MSSA strains. This finding was in agreement with the findings of Shore et al. (2011) and Le Maréchal et al. (2011) in sheep and human isolates respectively. The ST1153 found in A15 local isolate was in agreement with a study of Enany et al. (2010) and Moneck et al. (2011a) which indicated that the PVL+MSSA had hallmark characteristics from PVL+MRSA strains in that it possessed a novel MLST (ST1009) with a single variant (ST1153) as well as double locus variants (ST1216) (Table 4, Figure 2).

**TABLE 4:** The sequence type and the colonal complex of local isolates of *Staphylococcus aureus* isolates.

Number	Strain	MRSA	MSSA	ST	CC
1	A11	-	+	15	5
2	A12	-	+	130	130
3	A13	-	+	130	130
4	A15	-	+	1153	1153

ST, sequence type; CC, colonal complex; MRSA, methicillin resistant *Staphylococcus aureus*; MSSA, methicillin susceptible *Staphylococcus aureus*.

## Conclusion

MLST genotyping is a useful way to classify *S. aureus* which can also provide a highly efficient molecular epidemiological investigation. Three sequence types (ST15, ST130 and ST1153) were obtained in this study from MSSA isolated from she-camel’s milk. Phylogenetic analysis revealed close similarity between ST15 and ST1153, while the other ST130 is located in a separate cluster of other sequence types. Further investigations using this approach are needed to make a clear molecular epidemiological survey on *S. aureus* from camel’s milk.
